# Qualità del liquido seminale e assenza di SARS-CoV-2 RNA nel seme dopo infezione COVID-19: uno studio prospettico, osservazionale e validazione dello SpermCOVID test

**DOI:** 10.1007/s40619-022-01055-y

**Published:** 2022-03-14

**Authors:** Rossella Mazzilli, Antongiulio Faggiano

**Affiliations:** Unità di Endocrinologia, Dipartimento di Medicina Clinica e Molecolare, Azienda Ospedaliera Universitaria Sant’Andrea, Sapienza Università di Roma, Roma, Italia

Commento a:

**Sperm quality and absence of SARS-CoV-2 RNA in semen after COVID-19 infection: a prospective, observational study and validation of the SpermCOVID test.**

**G.G. Donders, E. Bosmans, J. Reumers, F. Donders, J. Jonckheere, G. Salembier, N. Stern, Y. Jacquemyn, W. Ombelet, C.E. Depuydt.**

**Fertil Steril (2021) 117(2):287–296**

La recente pandemia di *Severe acute respiratory syndrome* *CoronaVirus type 2* (SARS-CoV-2), responsabile della malattia *COronaVIrus Disease* 19 (COVID-19), ha sollevato diverse preoccupazioni nell’ambito della medicina della riproduzione [1]. Il virus colpisce la cellula ospite, principalmente lo pneumocita, legando il recettore rappresentato dall’enzima di conversione dell’angiotensina 2 (ACE2), per il quale mostra un’elevata affinità. L’espressione di tale enzima è stata riscontrata in numerosi tessuti umani, tra cui quello testicolare. Inoltre, si è ipotizzato che il virus possa superare la barriera emato-testicolare e causare un danno diretto allo spermatozoo. In letteratura, a favore di questa teoria, alcuni autori hanno descritto la presenza del virus SARS-CoV-2 nel liquido seminale di pazienti infetti [2]; tuttavia, alcuni errori metodologici sembrerebbero aver influenzato la validità dei risultati della ricerca. Infatti, la “sterilità” durante la raccolta del liquido seminale potrebbe essere stata inficiata da una contaminazione del contenitore attraverso *droplets*. Studi successivi non infatti hanno rilevato RNA virale nel liquido seminale di soggetti infetti con tampone naso-faringeo positivo [3].

Nell’ambito di tale scenario, un gruppo di ricercatori belgi ha recentemente condotto questo studio con lo scopo di: 1) determinare se SARS-CoV-2 potesse attraversare la barriera emato-testicolare, validando un sistema per la ricerca dell’RNA nel seme dopo l’infezione; 2) valutare l’impatto negativo dell’infezione sui parametri seminali, nonché sulla frammentazione del DNA (valutata mediante *Sperm chromatin structure assay*, SCSA) e sulla presenza di anticorpi anti-spermatozoo (studiati mediante *mixed erythrocyte-spermatozoa antiglobulin reaction*, MAR Test); 3) correlare le eventuali alterazioni seminologiche con il titolo anticorpale sierico (anticorpi anti SARS-CoV-2). Sulla base di tale proposito, i ricercatori hanno studiato 120 soggetti, con età compresa tra i 18 e i 70 anni con infezione COVID-19, risultati positivi al test per la ricerca di SARS-CoV-2 mediante tampone rinofaringeo in un periodo compreso tra una settimana e 6 mesi precedenti al momento dell’arruolamento.

L’RNA virale, analizzato mediante *reverse transcriptase-polymerase chain reaction* (RT-PCR), tecnica definita dagli autori come “SpermCOVID test”, non è stato rilevato in nessun campione seminale. Gli autori hanno tuttavia osservato una riduzione della motilità progressiva nel 60% dei casi a meno di un mese dell’infezione, nel 37% dei casi dopo 1–2 mesi e nel 28% dei casi dopo più di 2 mesi. Anche la concentrazione nemaspermica è risultata ridotta nel 37% dei casi valutati a meno di un mese dall’infezione, nel 29% dei casi tra 1 e 2 mesi e nel 6% dei casi dopo più di 2 mesi. Inoltre, a breve distanza dall’infezione, il tasso di frammentazione del DNA nemaspermico risultava più elevato. Tuttavia, né la gravità dell’infezione COVID-19, né la presenza di febbre correlavano con le caratteristiche seminali. Al contrario, emergeva una correlazione con un elevato titolo anticorpale (principalmente IgG SARS-CoV-2 contro la proteina “*Spike*” S1). Infine, non sono state osservate variazioni significative riguardanti la morfologia nemaspermica e il MAR-test.

Gli autori concludono che, seppur in assenza di evidenze riguardanti il passaggio del virus attraverso la barriera emato-testicolare, la qualità seminale dopo l’infezione COVID-19 può essere compromessa, e il tempo stimato per il recupero è di circa 3 mesi, ma gli autori sottolineano l’importanza di ulteriori studi di follow-up.
